# Bœnninghausen’s Aphorisms

**Published:** 1888-01

**Authors:** 


### BŒNNINGHAUSEN'S "APHORISMS."

(Translated by Wm. P. Wesselhœft, M. D., for the "Organon Society," Boston.)

The cause of all diseases lies in an internal, immaterial, purely dynamic change (*Verstimmung*) of the vital force, which may affect only single organs or extend itself to the entire organism. If heterogeneous or corrupt substances make their appearance, they should be regarded merely as products caused by the disturbance of the healthy vital force, but never looked upon as the first cause of disease. The local expulsion or dislodgment of such products can never restore the organism to its healthful action.

These natural diseases have a prototype in the action of those substances which we call medicine, in contradistinction to nutriments. These have a similar, purely dynamic power to so derange the vital forces as to produce a sick-making effect, closely resembling the natural diseases. The mysterious cause of this power to produce symptoms is and will remain as inscrutable to us as the knowledge of nature and essence of diseases.

It is an accepted truth, confirmed by repeated experiences, but not capable of proof by reasoning, that medicines possess the power to cure certain diseases. When the question is asked: Under what conditions does this occur? the two schools give opposite answers. The allopath will insist that it was by the action of contraries, the homœopath that the cure results according to the law of similars. Nevertheless, both schools are united in this: Recoveries can only occur when the medicine favorably affects the *vital force;* without this favorable effect, and subsequent *reaction*, all medicines will prove useless.

In this ever-present quality of reaction of the vital force we homœopaths recognize the foundation upon which rests the primary and secondary action of drugs. The primary action is the one which imparts its immediate sick-making qualities directly to and upon the living organism. The secondary action is that counter-action (reaction) of the living organism against the attacks of the primary action. These two actions are in direct opposition to each other, although they are the combined products of both the dynamic force of life and the dynamic force of the medicine. In this antagonism with each other they present differences which an experienced eye can readily detect.

The completed cure of a disease is the direct result of the secondary action, in which the living and constantly reacting organism is gradually and constantly attaining the upper hand in this strife with the medicine, till the natural disease has been conquered and annihilated, and thereby health restored.

From what has just been said, it becomes manifest that the homœopath should be exceedingly cautious not to disturb this strife between the primary and the reactive (secondary) action, and not to force upon the organism new and repeated doses, only to heighten and prolong the strife. According to our experience, nothing is more dangerous and harmful than *impatience.* He will never regret patient waiting so long as he knows—and which he should know by his knowledge of the characteristics of the medicine—that this strife is still in progress, unless he recognizes with certainty such a change in the indications which calls for another remedy. Should this inducement or necessity of giving another remedy occur (which, however, should not often happen), the surest criterions and cautions are known to us, whereby we may avoid harmful haste or a neglectful loss of time.

The time of awaiting the issue of the primary action of a drug varies exceedingly according to the nature and duration of the disease. In acute diseases, like cholera, the time may be measured by minutes, while in chronic diseases many weeks may ensue before the curative secondary action will become evident. It is in the old, tedious chronic diseases in which the greatest mischief is caused by too early repetitions or too hasty an interference with another remedy; and this mischief is not easily remedied, and is invariably followed by loss of time.

This is the cliff upon which the beginning homœopath is most usually wrecked, especially if he has too long served under the flag of allopathy.

